# Neurocognitive function and associations with mental health in adults born preterm with very low birthweight or small for gestational age at term

**DOI:** 10.3389/fpsyg.2022.1078232

**Published:** 2023-01-18

**Authors:** Siri Weider, Astrid M. W. Lærum, Kari Anne I. Evensen, Solveig Klæbo Reitan, Stian Lydersen, Ann Mari Brubakk, Jon Skranes, Marit S. Indredavik

**Affiliations:** ^1^Department of Psychology, Faculty of Social and Educational Sciences, Norwegian University of Science and Technology, Trondheim, Norway; ^2^Department of Clinical and Molecular Medicine, Faculty of Medicine and Health Sciences, Norwegian University of Science and Technology, Trondheim, Norway; ^3^Children’s Clinic, St. Olavs Hospital, Trondheim University Hospital, Trondheim, Norway; ^4^Unit for Physiotherapy Services, Trondheim Municipality, Trondheim, Norway; ^5^Department of Physiotherapy, Oslo Metropolitan University, Oslo, Norway; ^6^Department of Mental Health, Faculty of Medicine and Health Sciences, Norwegian University of Science and Technology, Trondheim, Norway; ^7^Department of Psychiatry, Division of Mental Health Care, St. Olavs Hospital, Trondheim University Hospital, Trondheim, Norway; ^8^Department of Pediatrics, Sørlandet Hospital, Arendal, Norway

**Keywords:** executive function, mental health, psychiatric symptoms and disorders, preterm birth, working memory, intelligence, follow-up, low birthweight

## Abstract

**Objectives:**

To assess neurocognitive function in adults born with low birthweight compared with controls and to explore associations between neurocognitive function and psychopathology in these groups.

**Methods:**

In this prospective cohort study, one group born preterm with very low birthweight (VLBW: birthweight <1,500 *g*, *n* = 53), one group born small for gestational age at term (SGA: birthweight <10th percentile, *n* = 63) and one term-born control group (birthweight ≥10th percentile, *n* = 81) were assessed with neurocognitive tests, diagnostic interviews, and self-report questionnaires at 26 years of age.

**Results:**

The VLBW group scored significantly below the control group on several neurocognitive measures, including IQ measures, psychomotor speed, verbal fluency, aspects of visual learning and memory, attention, social cognition, working memory and fine motor speed. The SGA group consistently scored at an intermediate level between the VLBW and the control group and had significantly lower scores than controls on Performance IQ and psychomotor speed, including switching. In the VLBW group, associations were found between lower spatial working memory and the presence of anxiety disorders, internalizing and attention problems, and autistic traits. Furthermore, lower Full scale IQ was associated with attention problems when adjusting for sex and parental socioeconomic status.

**Conclusion:**

Adults born preterm with VLBW or born term SGA displayed neurocognitive difficulties. Spatial working memory was associated with difficulties with attention, anxiety, and social function of VLBW adults. The finding and its clinical applicability should be further explored.

## 1. Introduction

Being born preterm with very low birthweight (VLBW, birthweight ≤1,500 *g*) or small for gestational age (SGA, birthweight <10th percentile) at term constitute important risk factors for later cognitive difficulties (Aarnoudse-Moens et al., 2009).

For individuals born with VLBW and/ or very preterm (born <32 weeks of gestation), there is evidence for impaired cognitive performance including lowered IQ scores, executive, and attentional difficulties in childhood (Mu et al., 2008; Aarnoudse-Moens et al., 2009; Murray et al., 2014; Breeman et al., 2015; Farajdokht et al., 2017; Córcoles-Parada et al., 2019), adolescence (Brydges et al., 2018; Twilhaar et al., 2020), and adulthood (Nosarti et al., 2007; Pyhälä et al., 2011; Eryigit Madzwamuse et al., 2015; Kroll et al., 2017; Eves et al., 2020; Perrone et al., 2020; Eves et al., 2021), and a dose–response-relationship between birthweight and IQ has been indicated (Gu et al., 2017).

Whether the described cognitive difficulties persist into adulthood has been a question of some controversy, and while an outgrowing of sequelae due to brain plasticity has been suggested (Curtis et al., 2002; Schneider et al., 2014), most researchers find support for lasting cognitive difficulties in individuals born preterm with VLBW (Pyhälä et al., 2011; Heinonen et al., 2013; Eryigit Madzwamuse et al., 2015; Kroll et al., 2017; Linsell et al., 2018; Stålnacke et al., 2019).

Being born SGA at term may indicate intrauterine growth restriction, which has been associated with later neurocognitive and neurodevelopmental abnormalities (Sharma et al., 2016; Sacchi et al., 2020) and poorer school performance (Lindström et al., 2017). In a review, de Bie et al. (2010) found that children and adolescents born SGA at term had lower IQ scores and more cognitive difficulties than controls. Likewise, Yi et al. (2016) reported lower verbal IQ scores in a group of term-born SGA children at 8–16 years of age. Difficulties on tests of attention and executive function in SGA individuals at 20 years of age was observed in a study by Suffren et al. (2017).

Poor cognitive function is found to be associated with lower real-life achievements in individuals born very preterm or with VLBW (Kroll et al., 2017). Furthermore, an increased risk of social difficulties and poor mental health is demonstrated for VLBW individuals (Kroll et al., 2018; Kajantie et al., 2019; Anderson et al., 2021; Ni et al., 2021). For extremely low birth weight (ELBW) individuals in middle childhood, frequently reported diagnoses include attention deficit/hyperactivity disorder (ADHD), autism spectrum disorders (ASD) and anxiety disorders (Johnson and Marlow, 2011; Anderson et al., 2021). Increased risk for mental health problems in adults born with low birthweight or born preterm was also found in a large meta-analysis by Pyhälä et al. (2017) as well as Anderson et al. (2021) and in register-based population studies (Nosarti et al., 2012; Class et al., 2014; Persson et al., 2020; Robinson et al., 2022).

In a previous follow-up of the present cohort in early adulthood (at 19–20 years of age), the preterm VLBW group had lower IQ scores than the control group (Løhaugen et al., 2010) and displayed problems related to memory (Aanes et al., 2015), attention and executive functions (Sølsnes et al., 2014; Østgård et al., 2016). The term born SGA group had lower IQ scores (Løhaugen et al., 2013) and modestly poorer scores than the control group on attention and executive functions, with more significant differences in the memory domains (Østgård et al., 2014). We found increased frequency of mental health problems at 14 and 20 years of age in the VLBW group, specifically anxiety disorders, attention problems and autistic traits (Indredavik et al., 2004; Lund et al., 2011), and furthermore increased frequency of psychiatric disorders and symptoms in both the preterm VLBW and the term born SGA group at 26 years of age (Lærum et al., 2017, 2019).

These reports raise the question whether lower cognitive reserve is associated with adult mental disorders in preterm VLBW, and term born SGA adults.

In this study, we aimed to assess neurocognitive function as well as its associations with the typical profile of psychiatric symptoms and diagnoses in adults born preterm at VLBW or born term SGA compared to controls, specifically anxiety/internalizing problems, inattention, and autistic traits. We expected adults born preterm with VLBW or term SGA to perform poorer than controls on tests of neurocognitive functions. Furthermore, we anticipated psychiatric symptoms/diagnoses to be associated with lower cognitive functioning.

## 2. Materials and methods

### 2.1. Study design

This is a prospective cohort study in which a preterm VLBW group, a term-born SGA group and a term-born control group were examined at 26 years of age. All participants were White and born between 1986 and 1988. The preterm VLBW subjects had been admitted to the Neonatal Intensive Care Unit (NICU) at Trondheim University Hospital, Norway. They were either born at this hospital or referred from local hospitals in the same health region. The term-born SGA and control participants were recruited from a multicenter study on causes and consequences of intrauterine growth restriction (Bakketeig et al., 1993; Vik et al., 1997). They were born to women residing in the same geographical region. Cognitive testing was performed by trained research assistants who were blinded to group adherence and previous findings and the results were discussed with an experienced clinical neuropsychologist on a regular basis. All questionnaires used at the 26-year follow-up were completed by the participants during project attendance. Further details of the study design have been published previously (Lærum et al., 2017).

### 2.2. Participants

**Figure 1:**
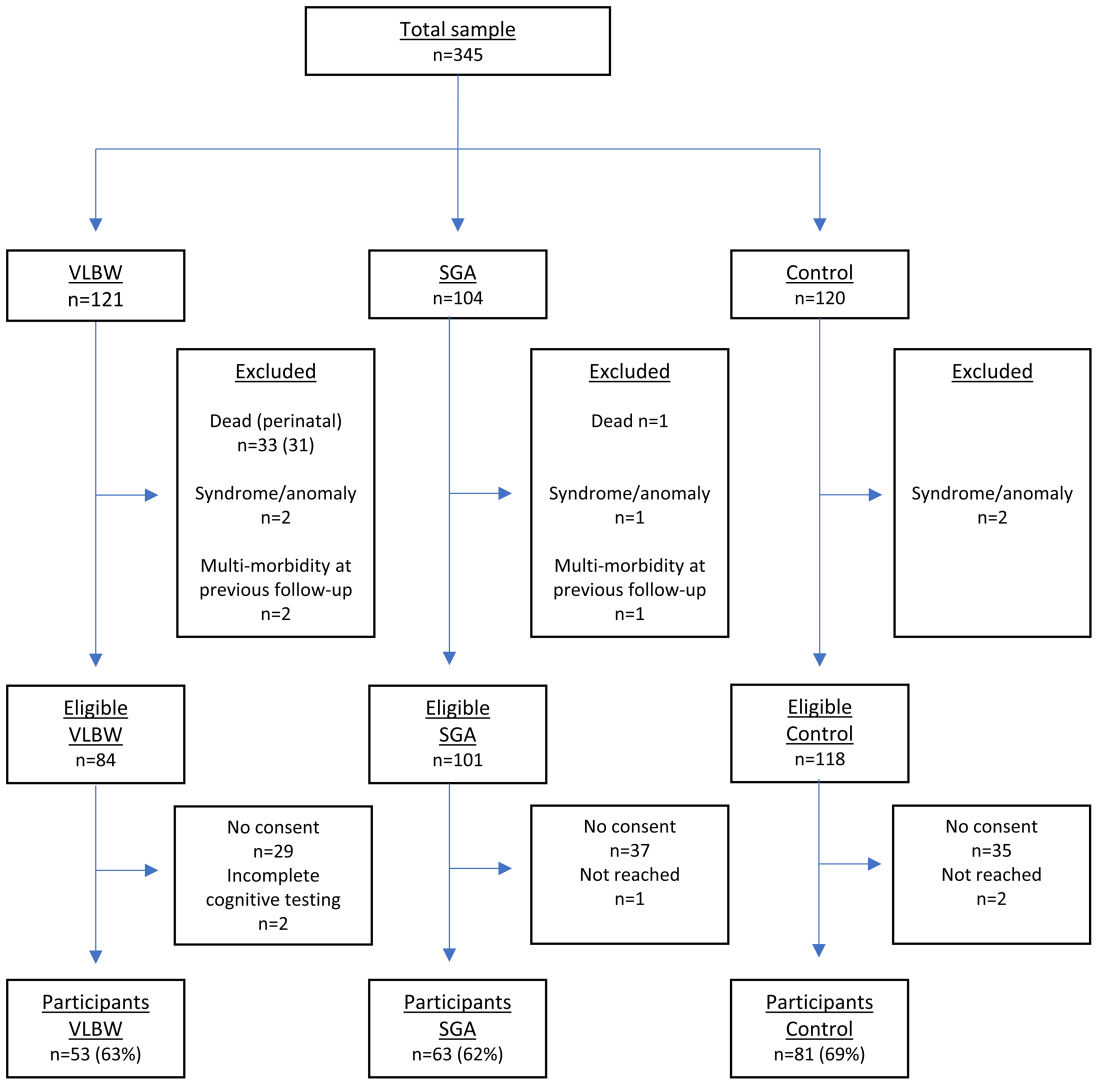
Flowchart of participation.

Figure 1 shows a flow chart of participation. The VLBW group was defined by BW ≤1,500 *g*, and all were born preterm. Of the 84 eligible for inclusion, 53 (62%) participated (26 males, 27 females) at mean age 26.4 (SD: 0.7). The SGA group was born at term with BW <10th percentile adjusted for gestational age, sex, and parity, according to Norwegian growth charts. Of the 101 eligible, 63 (62%) participated (32 males, 31 females) at mean age: 26.5 (SD: 0.5). The control group was born at term with BW ≥10th percentile. Of the 118 eligible, 81 (69%) participated (37 males, 44 females) at mean age 26.5 (SD: 0.5).

### 2.3. Cognitive measures

The participants completed a broad battery of conventional neuropsychological tests, including the Wechsler Abbreviated Scale of Intelligence (WASI) (Wechsler, 1997b), the Logical Memory subtest from Wechsler Memory Scale–3rd Edition (WMS-III) (Wechsler, 1997a), the Trail Making Test (TMT) and Verbal Fluency Test from Delis–Kaplan Executive System (D-KEFS) (Delis et al., 2001), and the Grooved Pegboard Test (Matthews and Klove, 1964). In addition, the following tests from the Cambridge Neuropsychological Test Automated Battery (CANTAB, Cambridge Cognition, 2006) were used: the induction tests Big/Little Circle and Motor Screening (results not shown), Attention Switching Task, Emotion Recognition Task, Intra-Extra-Dimensional Set Shift, Paired Associates Learning, Rapid Visual Information Processing, Spatial Working Memory, and Stockings of Cambridge. Results from some of the memory and working memory tests are published previously (Aanes et al., 2020) An overview of the neuropsychological tests is given in Appendix 1.

### 2.4. Mental health measures

To compare neurocognitive functions with mental health, we wanted to explore specific associations with the occurrence of anxiety/ internalizing problems, attention problems, and autistic traits. Psychiatric diagnoses were assessed using the Mini-International Neuropsychiatric Interview; M.I.N.I. Plus Norwegian version (MINI) (Sheehan and Lecrubier, 1994). The assessments were performed by trained clinicians and the main results are published previously (Lærum et al., 2017, 2019). In this paper, we include the current prevalence of anxiety disorders. The Achenbach System of Empirically Based Assessment, Adult Self-Report (ASEBA ASR) (Achenbach and Rescorla, 2003) is a self-administered questionnaire covering adaptive functioning and problems during the last 6 months. We used raw scores for Internalizing Problems (Anxious/Depressed, Withdrawn, and Somatic Complaints) and Attention Problems. The Autism-Spectrum Quotient (AQ) (Baron-Cohen et al., 2001) is a self-administered questionnaire consisting of 50 items assessing five domains of autism spectrum traits, of which we used the Sum score. For all measures, a higher score indicates more problems/mental health symptoms.

Parental socioeconomic status (SES), ranging from 1 (low) to 5 (high), had been calculated at 14 years (supplemented at 19 years) according to Hollingshead’s two-factor index of social position (Hollingshead, 1958), based on parents’ education and occupation. Parental SES was available for 45/53, 52/63, and 69/81 participants in the VLBW, SGA, and control group, respectively.

### 2.5. Statistical analysis

Outcome measures in the three groups were compared using linear regression with group as three category covariate. To preserve the familywise error rate due to comparisons between three groups, we reported the maximum *p*-values of the three groups comparison and pairwise comparisons (Lydersen, 2021). Associations between cognitive function and psychiatric symptoms were analyzed using linear regression, with ASEBA ASR Internalizing, ASEBA ASR Attention Problems and AQ Sum score as dependent variables. Associations between cognitive function and psychiatric disorders were analyzed by logistic regression with M.I.N.I. plus Anxiety Disorders as the dependent variable. Cognitive variables were entered one at a time and were selected to represent each of the neurocognitive domains; IQ total score (WASI); Verbal memory (WMS-III, Logical Memory, delayed); Psychomotor speed (TMT, Number Sequencing); Working memory (Spatial Working Memory); Mental flexibility/shifting (TMT, Number-Letter Switching); Social cognition (Emotion Recognition Task). These regression analyses were conducted with cognitive function and group and their interaction as covariates. All regression analysis were conducted unadjusted and adjusted for the possible confounders sex and parental SES in available case analyses. Ninety-five percent confidence intervals (CI) are reported where relevant. To provide some protection against false positive results due to multiple hypotheses, the two-sided significance level was set to 0.01.

### 2.6. Ethics statement

The study protocol for the 26-year follow-up was approved by the Regional Committee for Medical and Health Ethics (23879). REK Central Norway and the study was completed in accordance with the Declaration of Helsinki, with written informed consent and a protocol for referral.

## 3. Results

### 3.1. Clinical characteristics and previous results

Clinical characteristics are presented in Table 1. Birthweight, gestational age, and head circumference at birth differed between groups by study design. Cerebral palsy (CP) had been diagnosed in two VLBW participants at a previous follow-up. Age at participation and parental SES were similar in the three study groups.

**Table 1:** Clinical characteristics and psychiatric diagnoses and symptoms at 26 years of age in the three study groups.

Characteristics	*n*	Preterm VLBW	Term SGA	Control
Clinical characteristics				
Birthweight, *g*, mean (SD)	53/63/81	1,230 (234)	2,940 (198)	3,717 (440)
Gestational age, weeks, mean (SD)	53/63/81	29.2 (2.6)	39.6 (1.2)	39.8 (1.2)
Head circumference at birth, cm, mean (SD)^a^	44/54/77	27.1 (2.4)	33.9 (1.1)	35.5 (1.1)
Male sex, no. (%)	53/63/81	26 (49)	32 (51)	37 (46)
Twins, no. (%) ^a^	53/63/81	10 (19)	0 (0)	0 (0)
IVH, no. (%) ^b^	52/63/81	6 (12)	–	–
Cerebral palsy, no. (%)	53/63/81	2 (4)	0 (0)	0 (0)
Parental SES, mean (SD) ^c^	45/52/69	3.5 (1.3)	3.6 (1.2)	3.9 (1.0)
Age at participation, years, mean (SD)	53/63/81	26.4 (0.7)	26.5 (0.5)	26.5 (0.5)
Psychiatric diagnoses and symptoms				
M.I.N.I. plus; Any Anxiety Disorder, no. (%)	50/61/80	14 (28)	12 (20)	7 (9)
ASEBA ASR Internalizing, mean (SD)	52/61/80	11.4 (11.2)	11.2 (10.8)	7.4 (6.9)
ASEBA ASR Attention Problems, mean (SD)	52/61/80	6.8 (4.8)	6.0 (5.0)	4.8 (4.5)
AQ Sum score, mean (SD)	52/58/77	15.0 (6.3)	14.3 (5.9)	12.5 (5.3)

VLBW, very low birthweight; SGA, small for gestational age; IVH, intraventricular hemorrhage; SES, socioeconomic status; MINI, Mini-International Neuropsychiatric Interview; M.I.N.I. Plus Norwegian version; ASEBA ASR, Achenbach System of Empirically Based Assessment, Adult Self-Report; AQ, Autism-Spectrum Quotient.

a Twins from 8 pairs.

b IVH grade 1: three participants; IVH grade 2: two; IVH grade 3: zero; IVH grade 4: one.

c Parental SES collected at 14 years for *n* = 42 VLBW, 50 SGA, and 67 control and supplemented at 19 years for *n* = 3 VLBW, 2 SGA, and 2 control.

Psychiatric diagnoses and symptoms relevant for the objectives of this paper are presented in Table 1.

### 3.2. Neurocognitive function

Although scoring close to the normative means, the VLBW group had lower mean scores on the WASI Full scale, Verbal and Performance IQ compared to the control group (Table 2). In addition, this group were slower than controls on all conditions of the TMT. Furthermore, the VLBW participants had a lower number of correct scores on the category- and switching total correct responses on the Verbal Fluency Test and were slower with both hands on the Grooved Pegboard Test. Four VLBW participants had WASI scores <70. When we excluded these and the two VLBW participants with CP, the results were similar, but weakened for the WASI verbal IQ and the WMS-III delayed retrieval and total recognition (data not shown).

**Table 2:** Cognitive tests at 26 years of age in the three study groups.

Neuropsychological test	Preterm VLBW		Term SGA		Control	
	*n*	Mean (SD)	*p*	Mean (SD)	*p*	Mean (SD)
WASI, scaled score						
Verbal IQ	53/63/81	98.3 (17.0)	**0.001**	103.9 (12.1)	0.145	107.3 (12.5)
Performance IQ	53/63/81	101.6 (18.6)	**<0.001**	110.4 (10.6)	**0.008**	116.4 (10.9)
Total IQ	53/63/81	100.1 (17.7)	**<0.001**	107.9 (10.6)	0.019	113.2 (11.3)
WMS-III, raw score						
Logical memory I (immediate, story A + B + B)	53/63/81	38.7 (10.4)	0.086	39.9 (9.7)	0.130	42.4 (9.7)
Learning curve	53/63/81	4.2 (2.8)	0.040	5.2 (3.3)	0.529	5.5 (2.9)
Logical memory II (delayed, story A + B)	53/63/81	24.6 (8.0)	0.022	26.4 (7.2)	0.145	28.3 (7.5)
Logical memory; Total recognition	53/63/81	25.1 (2.9)	0.033	25.9 (2.4)	0.428	26.3 (2.3)
TMT, seconds to complete						
1. Visual scanning	53/63/81	24.0 (8.3)	**0.001**	22.8 (6.0)	**0.009**	20.0 (5.2)
2. Numbers	53/63/81	38.1 (17.2)	**<0.001**	33.4 (14.9)	**0.003**	26.8 (6.9)
3. Letters	52/62/81	42.9 (23.0)	**<0.001**	35.0 (12.6)	0.020	29.0 (9.8)
4. Letter-number switching	52/62/81	92.4 (43.1)	**<0.001**	77.2 (24.2)	**0.007**	64.4 (16.1)
5. Fine motor speed	53/63/81	26.9 (9.9)	**<0.001**	22.3 (5.7)	0.412	21.4 (5.6)
Verbal Fluency, raw score						
Letter, total correct	52/63/81	39.3 (10.3)	0.039	42.5 (11.4)	0.297	44.5 (12.0)
Category, total correct	53/63/80	43.5 (9.3)	**0.002**	45.8 (9.8)	0.029	49.5 (10.5)
Switching, total correct responses	53/63/81	13.3 (2.2)	**0.008**	13.9 (2.8)	0.056	14.7 (2.8)
Switching, total switching accuracy	53/63/81	12.5 (2.3)	0.011	13.0 (2.8)	0.057	13.9 (2.8)
Grooved egboard Test, seconds to complete						
Dominant hand	52/63/81	69.4 (24.3)	**<0.001**	61.9 (10.4)	0.174	58.4 (8.6)
Nondominant hand	53/63/81	79.6 (36.8)	**<0.001**	68.8 (9.5)	0.147	63.8 (9.0)

*p*-values vs controls. Mean (SD) are descriptive statistics. VLBW, very low birthweight; SGA, small for gestational age; WASI, Wechsler Abbreviated Scale of Intelligence; IQ, intelligence quotient; WMS-III, Wechsler Memory Scale –III; TMT, Trail Making Test. Values in bold indicate statistical significance.

On the CANTAB, the VLBW group had longer Attention Switching Task median correct latency, and lower Emotion Recognition Task total number of correct responses and percent correct, compared to the control group (Table 3). They also had more Paired Associates Learning total errors adjusted, lower scores on Rapid Visual Information Processing A’ and higher numbers of Spatial Working Memory between errors. The results were weakened when excluding participants with CP and/or IQ below 70, and only the Paired Associates Learning total errors adjusted and the Rapid Visual Information Processing A’ remained significant (data not shown).

**Table 3:** CANTAB outcome measures at 26 years of age in the three study groups.

		Preterm VLBW		Term SGA		Control
Test variable	*N*	Mean (SD)	*p*	Mean (SD)	*p*	Mean (SD)
AST median switching cost	52/63/81	−71.8 (112.1)	0.468	−75.1 (84.3)	0.468	−56.9 (90.2)
AST median congruency cost	52/63/81	84.4 (67.5)	0.985	76.2 (65.6)	0.748	84.2 (72.4)
AST median correct latency	52/63/81	775.2 (178.2)	**0.004**	684.0 (172.2)	0.764	692.0 (133.0)
AST total correct trials	52/63/81	145.0 (18.4)	0.016	149.8 (11.5)	0.401	151.6 (9.0)
AST total commission errors	52/63/81	1.1 (5.6)	0.135	0.1 (0.5)	0.954	0.1 (0.3)
AST total omission errors	52/63/81	2.4 (3.6)	0.018	1.1 (1.9)	0.786	1.3 (2.3)
ERT percent correct	52/63/81	63.2 (9.7)	**<0.001**	68.2 (9.2)	0.568	69.1 (8.2)
ERT median overall response latency	52/63/81	1639.7 (637.7)	0.164	1550.5 (321.2)	0.427	1492.5 (338.9)
ERT total number of correct responses	52/63/81	113.8 (17.4)	**<0.001**	122.8 (16.5)	0.568	124.3 (14.8)
IED total errors adjusted	53/63/81	31.8 (35.4)	0.135	24.9 (27.9)	0.492	21.5 (29.1)
IED EDS errors	53/63/81	10.1 (10.8)	0.358	8.4 (9.5)	0.650	7.7 (9.2)
PAL total errors adjusted	53/63/81	14.3 (23.7)	**<0.001**	6.4 (7.3)	0.584	5.2 (6.0)
RVP A’ prime	51/63/81	0.9 (0.1)	**0.002**	0.9 (0.1)	0.180	0.9 (0.01)
RVP median latency	51/63/81	401.4 (125.5)	0.341	374.6 (62.6)	0.491	385.4 (90.7)
SOC problems solved in minimum moves	53/63/81	9.4 (2.2)	0.019	10.1 (1.5)	0.592	10.3 (1.5)
SOC initial thinking time, 5 moves	53/63/80	13392.6 (8705.8)	0.973	13775.2 (9729.0)	0.970	13448.08.2 (9341.5)
SOC subsequent thinking time, 5 moves	53/63/80	810.8 (1239.7)	0.140	474.3 (644.0)	0.503	575.7 (790.9)
SWM between errors	53/63/80	28.5 (23.7)	**<0.001**	14.8 (13.8)	0.787	14.1 (14.9)
SWM strategy	53/63/80	32.8 (6.3)	0.071	30.1 (7.3)	0.823	30.3 (6.8)

*p*-values vs controls. Raw scores. Mean (SD) are descriptive statistics. VLBW, very low birthweight; SGA, small for gestational age; CANTAB, Cambridge Neuropsychological Test Automated Battery; AST, Attention Switching Task; ERT, Emotion Recognition Task; IED, Intra-Extra Dimensional Set Shift; PAL, Paired Associates Learning; RVP, Rapid Visual Information Processing; SOC, Stockings of Cambridge; SWM, Spatial Working Memory. Values in bold indicate statistical significance.

The SGA scored close to the normative means on all IQ measures, but below the controls on the WASI Performance IQ (Table 2). They performed at a slower speed than the controls on the following conditions of the TMT: visual scanning, numbers, and letter-number switching. The scores on the WMS-III, the Verbal Fluency Test, the Grooved Pegboard Test, and the CANTAB scores did not differ significantly from controls (Table 3).

Adjustments for sex and parental SES in available case analysis (*n* = 45 VLBW, *n* = 52 SGA, *n* = 69 Controls) did not change the results substantially for the VLBW group but decreased the difference between the SGA group and the controls on all measures as shown in (Supplementary Tables S1, S2).

### 3.3. Association between cognitive function and psychiatric diagnoses and symptoms

In the VLBW group, higher score on Spatial Working Memory between errors were associated with higher odds for anxiety disorders, and positively associated with ASEBA ASR Internalizing and Attention Problems and the AQ Sum score (Table 4). A negative association between WASI Full scale IQ and Attention Problems was present after adjusting for sex and Parental SES in the available case analysis (Supplementary Table S3). The results were mainly unchanged when we excluded participants with CP and/or IQ below 70 (data not shown).

**Table 4:** Logistic or linear regression with mental health as the dependent variable, and cognitive function and study group and their interaction as covariates.

	M.I.N.I. plus Anxiety Disorder		ASEBA ASR Internalizing		ASEBA ASR Attention Problems		AQ sum score	
Neuropsychological test	OR (95% CI)	*p*	B (95% CI)	*p*	B (95% CI)	*p*	B (95% CI)	*p*
Preterm VLBW, *n* = 52								
WASI, total IQ	0.962 (0.927 to 0.999)	0.045	−0.14 (−0.29 to 0.02)	0.079	−0.08 (−0.15 to −0.001)	0.047	−0.08 (−0.17 to −0.01)	0.077
WMS-III, LM II (delayed)	0.969 (0.896 to 1.049)	0.438	−0.01 (−0.34 to 0.32)	0.951	−0.07 (−0.23 to 0.10)	0.434	−0.11 (−0.31 to 0.09)	0.290
TMT, number sequencing	1.003 (0.969 to 1.039)	0.866	0.08 (−0.14 to 0.16)	0.918	0.06 (−0.02 to 0.13)	0.125	0.04 (−0.05 to 0.14)	0.354
SWM, between errors	1.042 (1.011 to 1.073)	**0.007**	0. 18 (0.07 to 0.29)	**0.001**	0.07 (0.02 to 0.13)	**0.007**	0.12 (0.06 to 0.19)	**<0.001**
TMT, letter-number switching	1.009 (0.993 to 1.024)	0.274	0.02 (−0.05 to 0.08)	0.626	0.01 (−0.02 to 0.04)	0.481	0.01 (−0.03 to 0.04)	0.811
ERT, total number correct	1.010 (0.974 to 1.048)	0.590	0.06 (−0.09 to 0.22)	0.415	−0.01 (−0.07 to 0.08)	0.849	0.01 (−0.08 to 0.10)	0.825
Term SGA, *n* = 61								
WASI, total IQ	0.958 (0.903 to 1.016)	0.151	−0.02 (−0.20 to 0.25)	0.844	0.03 (−0.08 to 0.14)	0.632	−0.11 (−0.25 to 0.02)	0.105
WMS-III, LM II (delayed)	0.951 (0.869 to 1.041)	0.274	−0.11 (−0.23 to 0.44)	0.537	−0.06 (−0.22 to 0.11)	0.495	−0.13 (−0.34 to 0.08)	0.210
TMT, number sequencing	1.004 (0.964 to 1.046)	0.858	−0.19 (−0.35 to −0.02)	0.026	−0.06 (−0.14 to 0.02)	0.148	−0.01 (−0.11 to 0.10)	0.906
SWM between errors	1.030 (0.987 to 1.074)	0.174	0.02 (−0.15 to 0.19)	0.838	0.002 (−0.08 to 0.09)	0.955	0.04 (−0.07 to 0.14)	0.472
TMT, letter-number switching	1.011 (0.986 to 1.037)	0.384	0.01 (−0.09 to 0.11)	0.851	0.01 (−0.04 to 0.06)	0.729	0.03 (−0.03 to 0.09)	0.376
ERT total number correct	1.005 (0.966 to 1.044)	0.817	−0.02 (−0.16 to 0.13)	0.818	−0.02 (−0.09 to 0.05)	0.575	−0.02 (−0.11 to 0.07)	0.641
Controls, *n* = 80								
WASI, total IQ	0.950 (0.887 to 1.017)	0.139	−0.08 (−0.26 to 0.11)	0.396	−0.05 (−0.14 to 0.04)	0.269	−0.06 (−0.18 to 0.05)	0.271
WMS-III, LM II (delayed)	0.963 (0.867 to 1.069)	0.477	0.00 (−0.28 to 0.28)	0.998	−0.04 (−0.18 to 0.10)	0.547	−0.09 (−0.27 to 0.09)	0.325
TMT, number sequencing	0.970 (0.857 to 1.097)	0.623	0.08 (−0.22 to 0.39)	0.593	0.18 (0.03 to 0.33)	0.018	0.16 (−0.03 to 0.35)	0.088
SWM between errors	1.035 (0.994 to 1.077)	0.095	0.13 (−0.01 to 0.27)	0.070	0.07 (−0.003 to 0.14)	0.060	−0.04 (−0.12 to 0.05)	0.391
TMT letter-number switching	0.992 (0.943 to 1.044)	0.766	0.04 (−0.09 to 0.17)	0.510	0.04 (−0.03 to 0.11)	0.226	−0.003 (−0.08 to 0.08)	0.939
ERT total number correct	1.010 (0.956 to 1.067)	0.730	0.02 (−0.13 to 0.16)	0.803	−0.001 (−0.07 to 0.07)	0.980	−0.06 (−0.15 to 0.03)	0.194

Unadjusted OR or B with 95% CI. VLBW, very low birthweight; SGA, small for gestational age; AQ, Autism-Spectrum Quotient; ASEBA ASR, Achenbach System of Empirically Based Assessment, Adult Self-Report; ERT, Emotion Recognition Task (CANTAB); WMS-III, Wechsler Memory Scale –III; LM, Logical Memory; TMT, Trail Making Test; WASI, Wechsler Abbreviated Scale of Intelligence; IQ, intelligence quotient; SWM, Spatial Working Memory (CANTAB). Odds Ratio (OR) or regression coefficient (B) with 95% Confidence Intervals (CI). *p*-values in bold indicate statistical significance.

In the SGA group and the control group, no associations were found between cognitive function and psychiatric diagnoses/ symptoms.

## 4. Discussion

In accordance with our expectations, adults born preterm with VLBW or SGA at term had several cognitive difficulties compared with controls. The VLBW group had reduced Full scale, Verbal and Performance IQ, lowered performance on aspects of visual learning, lowered psychomotor speed, poorer performance on tests measuring attention switching, verbal fluency, spatial working memory, visual processing, social cognition, and fine motor function. The SGA group had reduced Performance IQ and psychomotor speed. Associations between neurocognitive function and mental health were present in the VLBW group, where spatial working memory was associated with the more frequent presence of anxiety disorders, and with higher levels of internalizing problems, attention problems and autistic traits. Furthermore, a lower Full scale IQ was associated with more attention problems when adjusting for sex and parental SES (available case analysis). For the SGA group and the control group, no associations were found between cognitive function and mental health.

The results on cognitive function in this study are in accordance with previous studies on adults born preterm at VLBW, which reported difficulties related to IQ, executive function, attention and visual memory (Pyhälä et al., 2011; Eryigit Madzwamuse et al., 2015), and support the notion that cognitive difficulties persist into adulthood, as also shown for IQ in a recent individual participant data meta-analysis (Eves et al., 2021). Weak spatial working memory was furthermore seen in a study by Suikkanen et al. (2021) on the Cogstate Groton Maze Learning Test, and the authors argue that these difficulties could be related to the smaller size of the hippocampi, which was also found in the present sample at age 19–20 (Aanes et al., 2015). Reduced hippocampi volume was, moreover, found for the present preterm born sample at 26 years of age, but without significant correlation between spatial working memory and reduced hippocampal subfield volumes (Aanes et al., 2020).

Furthermore, the present study extends the current knowledge base on cognitive function in individuals with VLBW by revealing difficulties in visual learning and memory, verbal fluency, and social cognition. Visual memory difficulties in the VLBW group might be related to poor working memory function and thus difficulties in the initial encoding of the material. Weak verbal fluency, although mainly indicating difficulties in executive function such as initiation and generation, might suggest verbal challenges in adult age.

Of importance is the finding that individuals born SGA at term showed some cognitive difficulties in adult age with lower Performance IQ scores and reduced psychomotor speed compared to controls. This extends the findings from Viggedal et al. (2004) who found a slight reduction in IQ scores in young adulthood in individuals born SGA at term compared to controls, and underlines the importance of devoting attention to cognitive function in individuals born SGA in adult age.

Social cognition in adults born preterm with VLBW or SGA at term has not previously been investigated using the CANTAB. As described, the VLBW group performed below the controls on this task and are thus less able to differentiate between rapidly presented emotional facial expressions. This finding fits within the previous reports of poorer social skills in adults born with VLBW (Montagna and Nosarti, 2016). However, as the images used in the emotion recognition task are presented very briefly, these results could in part be due to problems with attention shifting and processing speed.

In the VLBW group, the associations between spatial working memory difficulties and symptoms belonging to the typical mental health profile consisting of elevated levels of attentional difficulties, symptoms of anxiety and poor social function (Johnson and Marlow, 2011) are especially noteworthy. Working memory is presumed to be important for everyday performance and a stronger predictor of academic achievement than IQ (Alloway and Alloway, 2010). Difficulties in spatial working memory was observed in very- and moderately preterm children at the age of 11 (Fitzpatrick et al., 2016), and in a NICU sample (of whom 78% were born preterm) measured with the CANTAB at age 9–14 (Curtis et al., 2002). Arthursson et al. (2017) found atypical neuronal activation in a group of very preterm adolescents during spatial working memory tasks, and the authors proposed a delayed maturation of the dorsolateral prefrontal cortex in the preterm group. Spatial working memory has also been found to be reduced in children with ASD (Zhang et al., 2020), and spatial working memory deficits are suggested to bring about social difficulties through problems in locating peers in context (Jiang et al., 2014). It is not unlikely that this might also affect social cognition in persons born at VLBW.

Furthermore, estimated IQ was associated with attention problems in the VLBW group after adjusting for sex and Parental SES. This is in accordance with findings from the general population and has also been suggested for extremely low birthweight individuals (Mathewson et al., 2017). The impact of IQ on psychopathology extends findings from a previous study on this VLBW cohort, where those exhibiting a psychiatric disorder at 15 and 19 years, or who developed psychopathology between these ages, had significantly lower IQ scores than VLBW participants without a psychiatric disorder at age 19 (Botellero et al., 2017). In the SGA group and the control group, however, there were no associations between cognitive function and mental health, despite increased prevalence of psychopathology in the SGA group at 26 years (Lærum et al., 2017). We need to bear in mind that the SGA group is heterogeneous, as some of them may be constitutionally small but otherwise healthy, and some may be growth restricted.

Taken together, our results regarding spatial working memory indicate a possible relationship between this function and anxiety, attention deficit and autistic traits in the VLBW group. The contribution of cognitive difficulties in general in a range of psychiatric disorders is well known (Millan et al., 2012), and individuals born preterm with VLBW may thus constitute a high-risk group for developing psychopathology. We speculate that cognitive difficulties affecting spatial working memory may imply reduced capacity to handle information and solve problems as well as reduced capacity to learn mental and social skills. Furthermore, altered brain development from pre- or perinatal exposures in VLBW individuals might result in vulnerability to both cognitive difficulties and psychopathology, though emerging or manifesting clinically at different ages. As cerebral MRI alterations are demonstrated in VLBW individuals (Volpe, 2009; Li et al., 2015), we speculate that SWM is specifically vulnerable to less optimal neurodevelopmental conditions in these individuals, and consequently, associations with mental health measures were found only in the VLBW group. Moreover, low SES and adverse environmental factors are found to be associated with being born with low birthweight (Blumenshine et al., 2010), as well as both cognitive difficulties and psychopathology (Gur et al., 2019). Overall, early detection of cognitive difficulties in help-seeking individuals born with low birthweight and concurrent psychopathology is important, as these individuals might require tailored therapeutic interventions targeting both functions.

We must bear in mind that the obstetric and neonatal care has improved considerably since the pre-surfactant period in the 1980s, when this VLBW cohort was born. Improved medical care, population health and socioeconomy have led to increased survival rates (Howson et al., 2013). Despite these advances, a meta-analysis displayed that extremely or very preterm children born in the antenatal corticosteroid and surfactant era show large deficits in intelligence, and no improvement in cognitive outcome was observed between 1990 and 2008 (Twilhaar et al., 2018). Another meta-analysis showed that children (4 to 17 years) born very preterm had medium to large deficits in three major cognitive domains: executive function, processing speed, and intelligence, with increasing severity with decreasing gestational age and/or birthweight (Brydges et al., 2018). Thus, even though our results may not be generalized to comparable gestational ages or birthweights in more recent cohorts, the results may still be relevant for those born with extremely low birthweight at the border of viability.

The strength of this study is the prospective design investigating both a preterm VLBW group and a term SGA group compared to a control group in adult age assessed with a comprehensive battery of neurocognitive tests. The mental health assessment was performed with validated instruments, and for the semi structured interview, means to ensure interrater reliability were applied. Apart from parental SES, our information is limited on environmental factors that may contribute to both cognitive development and mental health. However, all participants have grown up in Norway with equal access to health care and schooling. Furthermore, the fact that we had measures of SES for only a reduced sample, limits the applicability of this adjustment. Nonetheless, we chose to adjust for SES when analyzing the associations between neurocognitive function and mental health, since mental health is found to be strongly associated with SES (McLaughlin et al., 2011). Moreover, since the participants exhibited cognitive difficulties, self-report might not be the most reliable method for detecting psychiatric disorders/symptoms. The use of an abbreviated intelligence test (WASI) is a further limitation. Compared to a follow-up of the same sample at age 19–20 years (Løhaugen et al., 2010, 2013), when the Wechsler Adult Intelligence Scale, 3rd Edition (WAIS-III) (Wechsler, 1997a) was used, the IQ scores increased substantially and beyond what we would have expected in all three groups. Since there do not seem to be a similar increase in test scores from 19 to 20 years of age to 26 years of age on other neuropsychological tests such as the WMS-III (Østgård et al., 2014; Aanes et al., 2015), this discrepancy likely indicates a difference in the two IQ tests rather than an actual increase in IQ, and this elevated IQ-score might have masked associations between cognitive impairments and mental health problems. The use of the CANTAB set in “clinical mode” might constitute another weakness of this study, since a ceiling effect might have occurred. Since we used data from a large number of tests, we chose to reduce the risk of making a Type 1 error by applying a *p*-value of.01 as significance level. The use of this strict significance level might, however, have increased our risk of making a Type 2 error. The relative low sample size might furthermore involve increased risk of making Type 2 errors.

In the present study, a broad neuropsychological test battery was used to examine the neurocognitive functions of adults born preterm with VLBW or term SGA. The VLBW group performed significantly poorer than the control group on tests measuring several cognitive domains, while the SGA group performed at a level between the VLBW group and the control group yet indicating that they may have mild and unrecognized cognitive challenges. For the VLBW group, the association between spatial working memory and difficulties with attention, anxiety, and social function should be further explored in research and taken into consideration in the treatment of such psychiatric problems in this group.

## Data availability statement

The datasets generated and/or analysed during the current study are not publicly available as permission has not been provided by participants nor the Ethical Committee. Requests to access the datasets can be directed to the corresponding author.

## Ethics statement

The studies involving human participants were reviewed and approved by the Regional Committee for Medical and Health Ethics (REK Central Norway). The participants provided their written informed consent to participate in this study.

## Author contributions

SW contributed to the analyses and interpretation of data, drafted the initial manuscript, revised, and approved the final manuscript as submitted. AL carried out the analyses and interpretation of data, drafted the initial manuscript, revised, and approved the final manuscript as submitted. SR took part in and supervised the psychiatric assessment, reviewed, and revised the manuscript and approved the final manuscript as submitted. KE participated in conceptualization and designing the study, contributed to the analyses, reviewed, and revised the manuscript and approved the final manuscript as submitted. SL supervised statistical analyses, reviewed, and revised the manuscript and approved the final manuscript as submitted. A-MB participated in conceptualization and designing the study, reviewed, and revised the manuscript and approved the final manuscript as submitted. JS participated in conceptualization and designing the study, reviewed, and revised the manuscript and approved the final manuscript as submitted. MI participated in conceptualization and designing the study, coordinated and supervised data collection, supervised the analyses and interpretation of data, and revised and approved the final manuscript as submitted. All authors contributed to the article and approved the submitted version.

## Funding

The study was supported by the Norwegian Research Council and the Joint Research Committee between St. Olavs Hospital and Faculty of Medicine and Health Sciences, NTNU. AMWL was funded by the Liaison Committee for education, research and innovation in Central Norway.

## Conflict of interest

The authors declare that the research was conducted in the absence of any commercial or financial relationships that could be construed as a potential conflict of interest.

## Publisher’s note

All claims expressed in this article are solely those of the authors and do not necessarily represent those of their affiliated organizations, or those of the publisher, the editors and the reviewers. Any product that may be evaluated in this article, or claim that may be made by its manufacturer, is not guaranteed or endorsed by the publisher.
