# Supplementary material for: Neurocognitive function and associations with mental health in adults born preterm with very low birthweight or small for gestational age at term
Source: Front Psychol. 2023 Jan 18;13:1078232. doi: 10.3389/fpsyg.2022.1078232 (PMC9890170; doi:10.3389/fpsyg.2022.1078232)
Supplement: Supplementary file 1 [file Data_Sheet_1.pdf]

Online Appendix. Neuropsychological domains, tests and descriptions

Cognitive domain[a]	Neurocognitive test	Description of the test
**Intelligence**	WASI (1)<br>- Full scale intelligence quotient<br>- Verbal Intelligence quotient<br>- Performance Intelligence quotient	A brief test of intelligence consisting of two verbal subtests (Vocabulary and Similarities) and two performance subtests (Block Design and Matrix Reasoning).
**Learning and memory**	Logical memory from WMS-III (2)<br>- Logical memory I: the immediate recall of stories A+ B+ B<br>- The learning curve in logical memory I<br>- Logical memory II: the delayed recall of stories A + B<br>- Recognition: Correct answers	A test of verbal episodic memory. During this test, two stories are read out for the respondent (story A is read once, and story B is read twice) and the respondent is asked to recall the stories both immediately and after a delay. The test also contains a recognition condition in which the respondent is asked 30 true or false questions about the content of the two stories.
	Paired Associates Learning (PAL) [b]<br>- Total errors adjusted	In this test of visual memory and learning, the respondent is shown several boxes of which an increasing number contains a pattern. The boxes open one by one for a brief period, and the respondent is asked to remember the position of the box(es) containing the pattern(s). After all boxes have been opened and closed, patterns corresponding to those hidden within the boxes are displayed in the middle of the screen. The respondent`s task is to indicate in which box this pattern was hidden. In the first condition, only one out of six boxes contain a pattern. In the very last condition, there is a total of eight boxes all containing patterns to be remembered.
**Executive function**	Verbal Fluency Test from D-KEFS (3)<br>- Letter fluency; number of correct words<br>- Category fluency; number of correct words<br>- Switching; number of correct words<br>- Switching: number of correct switches	A test of verbal ability and executive control. The respondent is asked to produce as many words as possible within one minute over several conditions; words starting on a certain letter (three trials; F, A and S); words belonging to certain semantic categories (two trials; animals/ male first names) and words belonging to two semantic categories alternately.
	Attention Switching Task (AST) [b]<br>- Total correct trials<br>- Total commission errors<br>- Total omission errors<br>- Latency: time from the stimuli is presented on the screen to response<br>- Switching cost<br>- Congruency cost	A test of cued attentional set-shifting abilities. The respondent is shown a series of arrows on either the right or the left side of the screen. The arrows are pointing towards either right or left. In the initial trials, the respondent is prompted, on screen, to indicate the direction of the arrow or the side of the screen on which the arrow is displayed. During the final 96 trials, the respondent has to indicate the side, or the direction of the arrows based on rapidly shifting cues on screen.
	Intra-Extra Dimensional Set Shift (IED) [b]<br>- Total errors adjusted<br>- Extra-dimensional stage (EDS)-errors	During the nine stages of this set-shifting task, two figures are presented on screen in each trial. The respondent`s task is to choose between these patterns and to learn the rule of which pattern is correct. In stage 1, the stimuli consist of two solid pink shapes of which one of them is the target. In stage 2 the target is reversed, and the other

		presented shape is the target. In stage 3 a line-figure is introduced beside the familiar shapes, but the target is still one of the two solid shapes. This is also the case in stage 4. However, in this stage, the line-figure is overlapping with the familiar shape. Stage 5 is a reversion of the rule learnt in stage 4 (within the familiar shapes). In stage 6 new solid shapes and line-figures are presented (the Intradimensional shift). However, one of the new solid shapes is still the target, with a reversal in stage 7. In stage 8, the line-figure and not the shape is the target (the extradimensional shift) with a reversal in stage 9. The test is terminated if the respondent fails to complete any stage in 50 trials.
	<u>Stockings of Cambridge (SOC)</u> [b]<br>- Problems solved in min. moves<br>- Mean initial thinking time (5 moves)<br>- Mean subsequent thinking time (5 moves)	In this test of visuospatial executive abilities and spatial working memory, the stimuli on the upper half of the screen consists of three balls placed in different positions in three hanging stockings that might holds 3, 2 or 1 ball respectively. Similar stimuli are also displayed at the lower half of the screen, but the balls are here positioned differently. The task is to rearrange the balls within the stockings in the lower half of the screen to resemble the pattern in the upper part
**Working memory**	<u>Spatial Working Memory (SWM)</u>[b]<br>- Between errors<br>- Strategy	In this test, a number of closed boxes appear on the screen. The respondent`s task is to open these boxes one by one to locate a hidden token. Only one box contains a token at any one time, and when this token is found, a new token is placed within one of the other boxes on the screen. With an increasing number of boxes, the respondent must keep track of which boxes have already been opened, and in which of the boxes a token has previously been found during each trial.
**Attention**	<u>Rapid Visual Information Processing (RVP)</u>[b]<br>- A´ prime<br>- Latency: reaction time on hits in blocks 5-7.	In this test the respondent is asked to identify given strings of numbers among rapidly presented numbers on the screen. In the training phase the string 3-5-7 is the target and separates from other numbers by being written in red and being underlined in yellow. The color and the underlining disappear over trials, and the string has to be identified solely by the numbers. In the second phase of the test, two additional strings of numbers must be identified
**Psychomotor speed**	<u>Trail Making Test from D-KEFS (3)</u><br>- Visual scanning<br>- Numbers sequencing<br>- Letter sequencing<br>- Number-letter switching[c]<br>- Motor speed[d]	This test consists of five different trials; visually scanning the stimuli page and marking all circles containing the number 3; connecting numbered circles in numerical order; connecting circles containing letters in alphabetical order; connecting circles containing numbers and letters alternately in numerical and alphabetical order (i.e. 1-A-2-B-3-C etc.) and connecting circles by tracing a pre-drawn dotted line between empty circles.

**Social cognition**	Emotion Recognition Task (ERT)[b]<br>- Total correct responses<br>- Percentage of correct responses<br>- Latency: time from presentation of the different emotional choices to response	In this task, computer generated images of a person displaying different emotions are presented on the screen. The 90 images are each shown for a total duration of 200 milliseconds. There are six different emotions displayed in various levels of intensity, and the respondent is prompted to identify these.
**Motor function**	The Grooved Pegboard Test (4)<br>- Dominant hand<br>- Non-dominant hand	In this task, the respondent is asked to insert a total of 25 pegs into slots on a board. Each peg has a key and needs to be rotated manually to fit. The task is completed twice; with the dominant and the non-dominant hand respectively

Notes: Abbreviations: WASI, Wechsler Abbreviated Scale of Intelligence; WMS-III, Wechsler Memory Scale 3rd Edition; D-KEFS, Delis-Kaplan Executive Function System; [a] The domains under which each test is listed is based on the test as a whole. There are sub measures within several tests measuring other domains. [b] Test from the Cambridge Neuropsychological Test Automated Battery (CANTAB)(5); [c] Measures executive functioning, specifically set-shifting; [d] Measures motor speed

1. Wechsler D. Wechsler Abbreviated Scale of Intelligence. San Antonio, TX: Harcourt Assessment; 1999.
2. Wechsler D. Wechsler Memory Scale-Third Edition. San Antonio, TX: The Psychological Corporation; 1997.
3. Delis DC, Kaplan E, Kramer J. Delis Kaplan Executive Function System. San Antonio: The Psychological Corporation; 2001.
4. Matthews CG, Klove H. Instruction manual for the adult neuropsychology test battery. Madison: University of Wisconsin Medical School; 1964.
5. Cambridge Cognition. Neuropsychological Test Automated Battery (CANTABeclipse) manual. Cambridge, UK: Cambridge Cognition Limited; 2006.
