# Supplementary material for: Neurocognitive function and associations with mental health in adults born preterm with very low birthweight or small for gestational age at term
Source: Front Psychol. 2023 Jan 18;13:1078232. doi: 10.3389/fpsyg.2022.1078232 (PMC9890170; doi:10.3389/fpsyg.2022.1078232)
Supplement: Supplementary file 2 [file Data_Sheet_2.pdf]

Table S1. Cognitive tests at 26 years of age in the three study groups adjusted for sex and parental socioeconomic status.

Neuropsychological test		Preterm VLBW		Term SGA		Control
	n	Mean (SE)	*p*	Mean (SE)	*p*	Mean (SE)
WASI, scaled score						
Verbal IQ	45/52/69	99.1 (1.96)	***.002***	105.0 (1.82)	.434	106.9 (1.59)
Performance IQ	45/52/69	102.1 (1.78)	***<.001***	111.8 (1.78)	.040	116.7 (1.55)
Total IQ	45/52/69	100.9 (1.86)	***<.001***	109.3 (1.73)	.106	113.0 (1.51)
WMS-III, raw score						
Logical memory I (immediate, story A+B+B)	45/52/69	40.0 (1.41)	.181	40.8 (1.31)	.352	42.4 (1.14)
Learning curve	45/52/69	4.3 (0.44)	.078	5.6 (0.41)	.594	5.3 (0.36)
Logical memory II (delayed, story A+B)	45/52/69	25.7 (1.07)	.065	27.2 (1.00)	.425	28.3 (0.87)
Logical memory; Total recognition	45/52/69	25.4 (0.37)	.049	26.2 (0.34)	.759	26.3 (0.30)
TMT, seconds to complete						
1 Visual scanning	45/52/69	23.7 (0.98)	***.005***	22.2 (0.91)	.077	20.1(0.79)
2 Numbers	45/52/69	36.7 (1.60)	***<.001***	31.0 (1.48)	.015	26.2 (1.29)
3 Letters	45/52/69	41.9 (2.36)	***<.001***	33.9 (2.18)	.098	29.1 (1.88)
4 Letter-number switching	45/52/69	89.3 (4.26)	***<.001***	73.7 (3.93)	.068	64.1 (3.40)
5 Fine motor speed	45/52/69	26.6 (1.04)	***<.001***	22.2 (0.96)	.386	21.1 (0.84)
Verbal Fluency						
Letter, total correct	44/52/69	40.3 (1.70)	.077	43.8 (1.56)	.863	44.2 (1.36)
Category, total correct	45/52/68	44.2 (1.50)	***.004***	46.8 (1.40)	.111	49.8 (1.23)
Switching, total correct responses	44/52/69	13.4 (0.39)	***.008***	13.9 (0.36)	.058	14.8 (0.31)

Switching, total switching accuracy	44/52/69	12.6 (0.39)	.014	13.0 (0.36)	.068	13.9 (0.32)
Grooved Pegboard Test, seconds to complete						
Dominant hand	44/52/69	64.9 (1.57)	***<.001***	60.5 (1.44)	.192	58.0 (1.26)
Nondominant hand	45/52/69	75.7 (2.12)	***<.001***	67.8 (1.97)	.080	63.2 (1.72)

Note. *p*-values vs controls. Values in bold indicate statistical significance. Mean (SE) scores are estimated from the linear regression adjusted for sex and parental socioeconomic status. Abbreviations: VLBW: very low birthweight; SGA: small for gestational age; SE; standard error WASI: Wechsler Abbreviated Scale of Intelligence; IQ: intelligence quotient; WMS-III: Wechsler Memory Scale –III; TMT: Trail Making Test.
