# Supplementary material for: Neurocognitive function and associations with mental health in adults born preterm with very low birthweight or small for gestational age at term
Source: Front Psychol. 2023 Jan 18;13:1078232. doi: 10.3389/fpsyg.2022.1078232 (PMC9890170; doi:10.3389/fpsyg.2022.1078232)
Supplement: Supplementary file 3 [file Data_Sheet_3.pdf]

Table S2. CANTAB outcome measures at 26 years of age in the three study groups adjusted for sex and parental socioeconomic status.

		Preterm VLBW		Term SGA		Control
CANTAB measures	n	Mean (SE)	*p*	Mean (SE)	*p*	Mean (SE)
AST median switching cost	44/52/69	-78.9 (14.46)	.096	-68.4 (13.25)	.243	-47.8 (11.57)
AST median congruency cost	44/52/69	81.9 (10.0)	.985	72.7 (9.16)	.748	81.8 (8.00)
AST median correct latency	44/52/69	767.1 (22.59)	***.010***	685.8 (20.70)	.830	691.7 (18.07)
AST total correct trials	44/52/69	144.9 (1.88)	***.008***	150.2 (1.72)	.616	151.3 (1.50)
AST total commission errors	44/52/69	1.3 (0.48)	.050	0.2 (0.44)	.894	0.1 (0.38)
AST total omission errors	44/52/69	2.3 (0.38)	.013	1.1 (0.35)	.901	1.1 (0.30)
ERT percent correct	44/52/69	63.7 (1.37)	***.008***	68.9 (1.26)	.800	68.5 (1.10)
ERT median overall response latency	44/52/69	1548.1 (53.10)	.349	1599.4 (48.66)	.076	1483.8 (42.49)
ERT total number of correct responses	44/52/69	114.7 (2.47)	***.008***	124.00 (2.26)	.800	123.2 (1.97)
IED total errors adjusted	45/52/69	27.3 (4.08)	.485	24.5 (3.78)	.867	23.6 (3.30)
IED EDS errors	45/52/69	9.6 (1.45)	.409	7.9 (1.34)	.925	8.1 (1.17)
PAL total errors adjusted	45/52/69	15.2 (2.14)	***<.001***	6.2 (1.99)	.697	5.1 (1.73)
RVP A' prime	43/52/69	0.9 (0.01)	.011	0.9 (0.1)	.632	0.9 (0.01)
RVP median latency	43/52/69	404.4 (14.96)	.462	376.7 (13.52)	.451	390.3 (11.82)
SOC problems solved in minimum moves	45/52/69	9.4 (0.26)	***.005***	10.1 (0.24)	.459	10.4 (0.21)
SOC initial thinking time, 5 moves	45/52/69	13248.9 (1380.94)	.948	14917.8 (1280.60)	.298	13132.4 (1125.78)
SOC subsequent thinking time, 5 moves	45/52/69	847.0 (140.30)	.144	442.5 (130.11)	.429	580.2 (114.38)
SWM between errors	44/52/68	27.9 (2.59)	***<.001***	14.3 (2.40)	.862	13.8 (2.11)
SWM strategy	44/52/68	32.5 (0.99)	.153	29.9 (0.92)	.583	30.6 (0.81)

Note. p-values vs controls. Values in bold indicate statistical significance. Abbreviations: VLBW: very low birthweight; SGA: small for gestational age; CANTAB: Cambridge Neuropsychological Test Automated Battery; SE: standard error; AST: Attention Switching Task; ERT: Emotion Recognition Task; IED: Intra-Extra Dimensional Set Shift; PAL: Paired Associates Learning; RVP: Rapid Visual Information Processing; SOC: Stockings of Cambridge; SWM: Spatial Working Memory.
