# Supplementary material for: Neurocognitive function and associations with mental health in adults born preterm with very low birthweight or small for gestational age at term
Source: Front Psychol. 2023 Jan 18;13:1078232. doi: 10.3389/fpsyg.2022.1078232 (PMC9890170; doi:10.3389/fpsyg.2022.1078232)
Supplement: Supplementary file 4 [file Data_Sheet_4.pdf]

Table S3. Logistic or linear regression with mental health as the dependent variable, and cognitive function and study group and their interaction as covariates, adjusted for sex and parental socioeconomic status. Odds Ratio (OR) or regression coefficient (B) with 95% Confidence Intervals (CI).

	M.I.N.I. plus Anxiety Disorder		ASEBA ASR Internalizing		ASEBA ASR Attention Problems		AQ sum score	
Neuropsychological test	OR (95% CI)	*p*	B (95% CI)	*p*	B (95% CI)	*p*	B (95% CI)	*p*
Preterm VLBW, n=44								
WASI, total IQ	0.961 (0.920 to 1.004)	.077	-0.17 (-0.33 to -0.003)	.046	-0.12 (-0.20 to -0.03)	***.006***	-0.13 (-0.23 to -0.03)	.013
WMS-III, LM II (delayed)	0.997 (0.909 to 1.093)	.942	-0.02 (-0.38 to 0.35)	.930	-0.13 (-0.32 to 0.06)	.168	-0.17 (-0.39 to 0.06)	.139
TMT, number sequencing	1.012 (0.973 to 1.051)	.556	0.06 (-0.10 to 0.23)	.442	0.10 (0.02 to 0.18)	.020	0.08 (-0.02 to 0.19)	.107
SWM, between errors	1.057 (1.018 to 1.097)	***.004***	0.22 (0.10 to 0.33)	***<.001***	0.10 (0.04 to 0.16)	***<.001***	0.14 (0.07 to 0.21)	***<.001***
TMT, letter-number switching	1.006 (0.989 to 1.024)	.476	-0.004 (-0.07 to 0.06)	.900	0.01 (-0.02 to 0.05)	.518	0.01 (-0.03 to 0.05)	.561
ERT, total number correct	1.013 (0.973 to 1.055)	.530	0.03 (-0.13 to 0.19)	.702	-0.02 (-0.10 to 0.06)	.681	0.01 (-0.09 to 0.10)	.810
Term SGA, n=51								
WASI, total IQ	0.927 (0.858 to 1.001)	.054	0.03 (-0.25 to 0.30)	.848	0.06 (-0.08 to 0.19)	.435	-0.17 (-0.34 to 0.004)	.055
WMS-III, LM II (delayed)	0.925 (0.837 to 1.023)	.129	0.12 (-0.26 to 0.49)	.541	-0.03 (-0.22 to 0.16)	.776	-0.16 (-0.40 to 0.08)	.189
TMT, number sequencing	1.054 (0.973 to 1.141)	.195	-0.32 (-0.64 to 0.01)	.054	-0.09 (-0.25 to 0.07)	.275	0.12 (-0.11 to 0.35)	.292
SWM between errors	1.045 (0.995 to 1.098)	.082	-0.03 (-0.22 to 0.16)	.032	-0.04 (-0.13 to 0.06)	.476	0.03 (-0.09 to 0.15)	.660
TMT, letter-number switching	1.027 (0.997 to 1.058)	.073	0.001 (-0.12 to 0.12)	.988	-0.01 (-0.07 to 0.06)	.860	0.02 (-0.05 to 0.09)	.562
ERT total number correct	1.003 (0.962 to 1.046)	.889	0.02 (-0.14 to 0.17)	.840	-0.01 (-0.09 to 0.08)	.885	-0.03 (-0.13 to 0.07)	.543
Controls, n=69								

WASI, total IQ	0.967 (0.896 to 1.044)	.390	-0.07 (-0.26 to 0.12)	.483	-0.03 (-0.12 to 0.07)	.606	-0.05 (-0.17 to 0.07)	.427
WMS-III, LM II (delayed)	0.966 (0.855 to 1.091)	.578	-0.05 (-0.35 to 0.25)	.735	-0.05 (-0.20 to 0.11)	.547	-0.07 (-0.26 to 0.12)	.467
TMT, number sequencing	0.982 (0.839 to 1.149)	.823	0.22 (-0.13 to 0.57)	.221	0.22 (0.04 to 0.39)	.014	0.12 (-0.10 to 0.34)	.278
SWM between errors	1.024 (0.978 to 1.072)	.311	0.11 (-0.03 to 0.25)	.135	0.06 (-0.01 to 0.13)	.115	-0.02 (-0.11 to 0.07)	.631
TMT letter-number switching	0.988 (0.930 to 1.049)	.694	0.06 (-0.08 to 0.19)	.390	0.04 (-0.04 to 0.10)	.327	-0.02 (-0.10 to 0.06)	.637
ERT total number correct	1.020 (0.955 to 1.089)	.559	0.001 (-0.15 to 0.15)	.993	0.01 (-0.07 to 0.09)	.788	-0.07 (-0.16 to 0.04)	.162

Note. p- values in bold indicate statistical significance. Abbreviations: VLBW: very low birthweight; SGA: small for gestational age; AQ: Autism-Spectrum Quotient; ASEBA ASR: Achenbach System of Empirically Based Assessment, Adult Self-Report; ERT: Emotion Recognition Task (CANTAB) WMS-III: Wechsler Memory Scale –III; LM: Logical Memory; TMT: Trail Making Test; WASI: Wechsler Abbreviated Scale of Intelligence; IQ: intelligence quotient; SWM: Spatial Working Memory (CANTAB).
